# Assessment of Biocontainment Efficacy and Flow Cytometric Impact of a Novel Platform in High Containment Laboratories

**DOI:** 10.1177/15356760251378149

**Published:** 2025-09-26

**Authors:** Melanie Cohen, Julie Laux, Iyadh Douagi

**Affiliations:** Flow Cytometry Section, Research Technologies Branch, National Institute of Allergy and Infectious Diseases, National Institutes of Health, Bethesda, MD, USA.

**Keywords:** high containment, aerosols, flow cytometry, biosafety, cell analysis, infectious disease

## Abstract

**Introduction:**

Flow cytometry remains one of the most central technologies with broad applications in immunology and infectious disease research. In the lack of compatible measures for pathogen inactivation, cytometric analysis of live cells derived from infectious samples classically poses exposure risks to both operators and environment. Enclosing a cell analyzer in a biosafety cabinet (BSC) could significantly improve safety margins for laboratory operations but has unknown impacts on instrument performance. Here, we describe and characterize the performance of a novel, BSC-housed analyzer for cytometric analysis of infectious samples within a biosafety level-3 laboratory.

**Methods:**

We investigated internal BSC and ultraviolet (UV) laser temperature over time. System stability was assessed using quality control particles with the BSC on and off. Aerosol generation was tested at four locations in normal and simulated failure modes using fluorescent Dragon Green beads.

**Results:**

Under these conditions, the temperature of the BSC and UV laser was stable during operations. The BSC did not significantly impact instrument performance or stability. Low amounts of aerosols were detected from vulnerable points in the system, and 15-fold reduction in aerosol generation was achieved with the introduction of a plate seal.

**Conclusions:**

Utilizing a tailored platform for cell analysis in high-containment laboratory, we demonstrate that BSC enclosure of the analyzer preserved operational performance of both units. Aerosol measurement studies revealed the importance of a BSC-contained instrument when investigating infectious samples. Our findings stress the need to accelerate efforts toward the integration of cytometry-based technologies in high-containment laboratories.

## Introduction

Flow cytometry is a powerful analytical tool for single-cell analysis that has been crucial in both clinical diagnostics and biomedical research for over four decades.^1^ Increased demand for this type of instrumentation has led to technological innovations, increased applications, and expanded frequency and diversity of samples analyzed.^1,2^ Typically, flow cytometers analyze biological material—some of which may be considered biohazardous and can foster known and unknown infectious microorganisms.^3^ Research programs focused on studying the World Health Organization and National Institutes of Health (NIH) categorized Risk Group 3 and 4 human and animal pathogens and have a public duty to safely and responsibly investigate these relevant organisms.^4^ These pathogens are of public health, biosecurity, and biodefense interest, particularly pertaining to prevention, vaccine, and post-exposure prophylaxis development.^5^ Pathogens within Risk Groups 3 and 4 need to be researched in preparation for novel disease outbreaks and potential acts of bioterrorism.^5^ Emerging pathogens of interest, such as H5N1 avian influenza A and monkeypox virus, are becoming an increasing threat to public health and safety, so the ability to appropriately characterize diseases, monitor pathogens, strategize treatment, and optimize testing is paramount.^6^ The combined risks of new threats, paired with the lingering presence of pathogens like SARS-CoV-2 (COVID-19), measles, and *Mycobacterium tuberculosis*, have contributed to a substantial increase in infectious sample analysis in both research and clinical laboratories, with an emphasis on rapid testing and community contact tracing.^6^ With this increased research and clinical interest, flow cytometry analysis of infectious samples can pose a potential exposure risk to both operators and the environment if the proper containment measures are not taken into consideration.^7^

Many pieces of laboratory equipment and procedures are known to generate aerosols. Centrifuges, vortexes, tissue homogenizers, and flow cytometers all carry a varying degree of risk for aerosol generation. Standard operating procedures, engineering controls, and personal protective equipment (PPE) have been developed to mitigate operator exposure risks.^8^ Flow cytometry carries an inherent risk to both the operator and environment, as proven by well-documented aerosol generation connected to procedures such as cell sorting.^7,9,10^ Cell sorters classically operate using high-pressure fluidics (10–70 pounds per square inch [psi]) and rapid vibrating devices for droplet generation, causing intentional aerosol production.^3^ The aerosolization of biohazardous material during cell sorting may release submicron infectious particles into the air for extended periods of time, which would increase the likelihood of exposure to laboratory personnel.^3^ Recognizing this risk during cell sorting procedures, the International Society for Advancement of Cytometry released safety guidance in 1997, 2007, and 2014.^3,9,11^ These biosafety guidelines recommend placing flow cytometry cell sorters within a biosafety cabinet (BSC) to reduce the risk of personnel coming into contact with aerosols during infectious cell sorting.^9,10^ By default, most cell sorters on the market today are housed within an integrated BSC often provided by the manufacturer.

In addition to the well-established biosafety guidelines to protect the user and environment from infectious aerosols, sorters have the additional safeguard of an aerosol management system (AMS).^9^ AMS units are validated operator protections that have been universally incorporated to cell sorter systems for the past 20 years. During operational sorting failure, infectious aerosols potentially breaching containment are captured with the AMS filtration system; therefore, the chance of exposure drastically decreases when in use.^12^ Methodology for testing cell sorting containment in the biosafety level 2 (BSL-2) and BSL-3 setting has been standardized and recommended to all flow cytometry laboratories operating with infectious agents.^12–14^ Prior to cytometer integration into a high-containment lab, considerations such as laboratory needs, potential pathogens of interest, biosafety restrictions, and personnel should be considered.^15^ When operating cytometers at a BSL-3 or higher, consistent containment testing and safety standardization protocols need to be thoroughly enforced and monitored to ensure personnel and environmental safety.^15^

Unlike cell sorters, benchtop analyzers operate under different sample delivery mechanisms and do not have integrated AMS. Typically, most analyzers do not utilize jet-in-air fluidics and operate under lower (2–10 psi) or no pressure, so there are fewer aerosolization opportunities. The risk of aerosol hazards from benchtop analytical flow cytometers has been previously discussed but not fully characterized.^16^ More recently, we demonstrated that under certain operational conditions analyzers can generate aerosols, such as operational failure at the sample loading port, vigorous automatic sample mixing, and incorrect waste management.^17^ The Biosafety in Microbiological and Biomedical Laboratories (BMBL) guidelines recommend that splash or aerosol-generating procedures of potentially infectious material be performed in a primary enclosure, such as a BSC, when operating at a BSL-2 or higher.^18^ This further supports the rationale to enclose analyzers in a BSC when analyzing high-risk material, but little concrete evidence is presently in the literature.^18^

There are established flow cytometry analysis protocols to mitigate risk with infectious samples, which are based on lab-specific risk assessments. Risk mitigation strategies include sample fixation, enhanced operator PPE, BSC enclosure, and enhanced decontamination procedures.^18^ Fixation is not an appropriate measure for all sample and analysis categories, but it is an actionable solution for analysis of some infectious samples and provides necessary risk-reduction for the operator and laboratory environment.^16^ Moreover, many functional assays require live cell analysis and therefore preclude fixation, such as calcium flux, cytotoxicity assays, apoptosis assays, and assessment of metabolic states and reactive oxygen species.^2,14,16^ In addition, some fluorescent proteins, autofluorescence, and cell-type-specific light scatter can be altered with fixation.^19,20^ If fixation is not possible due to experimental constraints, analyzing live, infectious samples via flow cytometry within a BSC is necessary for required safety standards. Enclosing flow cytometry analyzers in a BSC has been proposed, but they have historically been too large for BSC enclosure.^16^ Technological advancements have allowed analyzers to occupy a smaller footprint, therefore allowing BSC enclosure to become feasible. Despite the growing need for high-containment flow cytometry, the necessity and practicality of infectious sample flow cytometry analysis within a BSC has not been fully addressed.

While fully enclosing an analyzer in a BSC is the recommended risk-mitigating action, characterizing and understanding the operational impact of the BSC on instrument function and data generation is critical. We hypothesize that fluctuating temperature and increasing vibration from the BSC may affect system performance over the course of an experiment. Here, we explore the setup, performance, data output, and containment of a customized benchtop analyzer fully enclosed within a Class II BSC in a BSL-3 laboratory. Our study is expected to serve as building blocks for modernized biosafety guidelines and laboratory risk assessments involving pathogenic cell analysis in a high-containment environment.

## Materials and Methods

### Laboratory and Instruments

All experiments were performed within a BSL-3 laboratory on the NIH main campus in Bethesda, MD. No infectious materials were used in the conduct of this study. All experiments were performed using a Beckman Coulter CytoFLEX LX equipped with 6 lasers and 22 channels, 20 fluorescent parameters, and 2 light scatter parameters (Beckman Coulter, Brea, CA) (Supplementary Figure S1). The CytoFLEX LX can analyze samples via tube and multi-well plate. Standard instrument quality control (QC) was performed per manufacturer’s recommended procedures and prior to any data collection.

**Figure 1.**
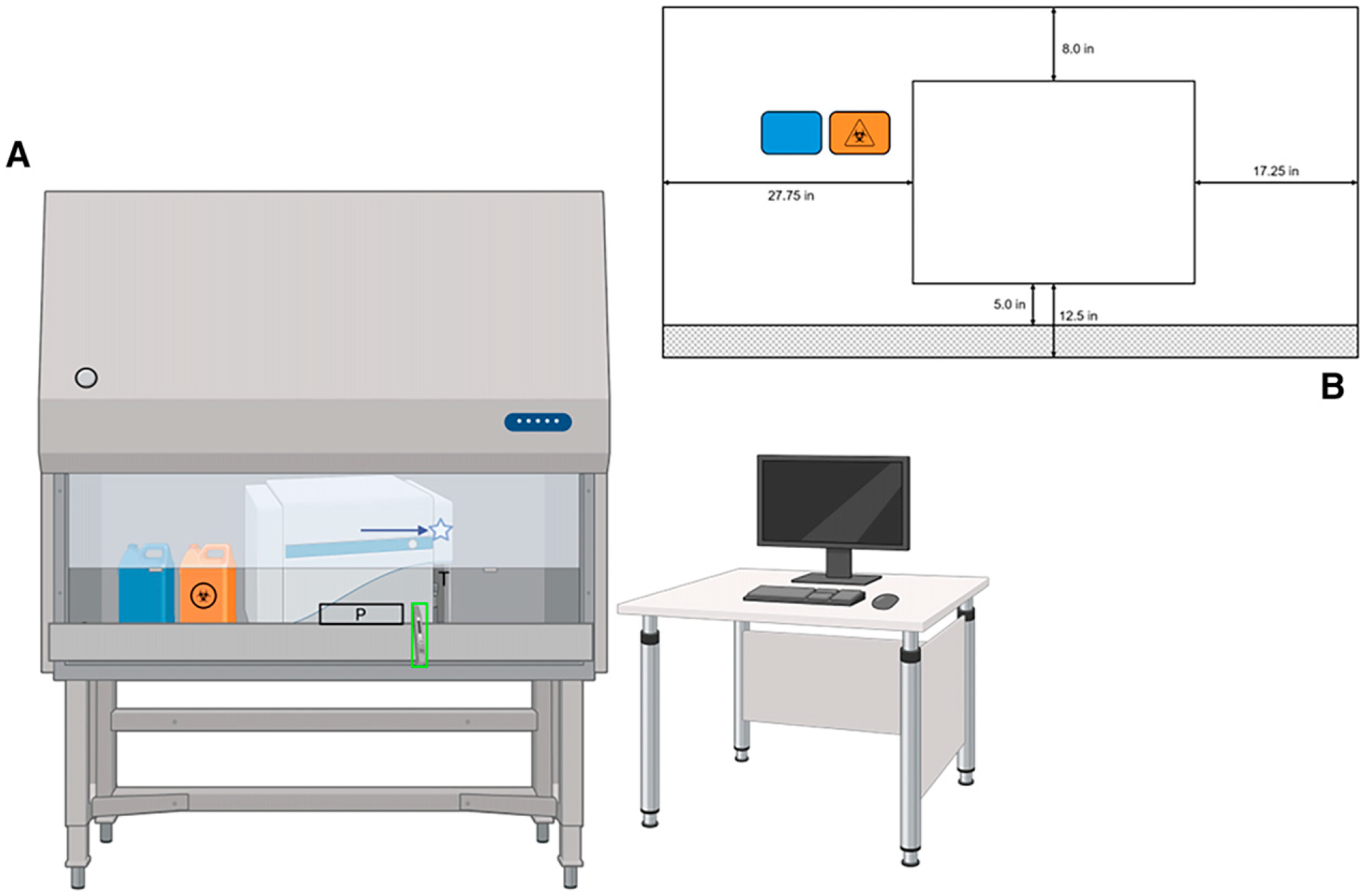
Diagram of cytometer setup in the BSL-3 laboratory. **(A)** The analyzer, sheath container (solid blue container), and waste container (solid orange container) are housed within the biosafety cabinet. Within the analyzer, T and P are the sample tube loader and plate loader, respectively. The lasers are represented by the blue arrow, and the interrogation point is represented by the star. The gray detector between P and T represents the temperature datalogger (outlined in green), which was in the BSC. The computer workstation sits outside the BSC. Other engineering controls not represented in this diagram include the laboratory under negative pressure. The operator wears PPE appropriate for the lab, agents, and manipulations. Due to security reasons, an actual photo of the analyzer used was not possible, so the figure was created in BioRender. Laux, J. (2025) https://BioRender.com/ktoizv4. **(B)** Instrument orientation within the BSC. The placement measurements are as follows: 17.25 inches from right side of instrument to right wall of BSC; 27.75 inches from the left side of the instrument to the left wall of the BSC; 8 inches from the back of the instrument to the back wall of the BSC; 5 inches from the front of the instrument to the perforated front grill; 12 inches from the front of the instrument to the sash; and 12.5 inches from the top of the instrument to the perforated aluminum diffuser (not shown). BSC, biosafety cabinet; BSL, biosafety level; PPE, personal protective equipment.

The flow cytometer was housed within a customized Class II BSC to accommodate the dimensions of the CytoFLEX LX and sheath and waste tanks and allow for access to the side and top panels of the cytometer (NuAire, Plymouth, MN). Dimensions and airflow specifications are shown in Supplementary Figure S2. A third-party contractor performed the certification (ENV Services, Hatfield, PA). Certification included the following tests: downflow velocity test, inflow velocity test, High-Efficiency Particulate Air filter leak test, airflow smoke patterns test, and electrical safety test. A diagram of the instrument setup in the BSL-3 laboratory is shown in Figure 1A. Within the BSC, the instrument sits right of center. The placement measurements are as follows: 17.25 inches from right side of instrument to right wall of BSC; 27.75 inches from the left side of the instrument to the left wall of the BSC; 8 inches from the back of the instrument to the back wall of the BSC; 12.5 inches from the top of the instrument to the perforated aluminum diffuser; 5 inches from the front of the instrument to the perforated front grill; and 12 inches from the front of the instrument to the sash (Figure 1B).

### Sample Preparation

Daily QC beads and infrared (IR) beads (Beckman Coulter, catalog# B53230 and catalog# C06147, Brea, CA) were used to measure signal stability. The daily QC beads were diluted in deionized water according to the manufacturer’s recommendations (Quality Biological, catalog# 118-162-131, Gaithersburg, MD). The IR QC beads are pre-diluted and were analyzed on the instrument directly from the vial per manufacturer’s recommendation. The multi-size Cytometer Setup and Tracking (CS&T) QC beads (Becton Dickinson, catalog# 655051, Franklin Lakes, NJ) were used to measure instrument stability. CS&T beads were prepared according to the manufacturer’s recommendation in 1× phosphate buffered saline (PBS) (Quality Biological, catalog# 114-058-131, Gaithersburg, MD). Dragon Beads (DB) with excitation at 480 nanometers (nm) and emission at 520 nm (1.0 μm, Bangs Laboratories, Fishers, IN) were prepared as a 1:100 dilution in 1× PBS and 0.5% Tween20 (Fisher Bioreagents, Fair Lawn, NJ) and used for aerosol testing.

### Temperature Logging

An Extech RHT10 temperature data logger (Extech Instruments, Nashua, NH) was placed within the BSC between the tube loader and plate loader to record the temperature inside the BSC (Figure 1A). The data logger recorded the temperature at one-minute intervals for the duration of the experiments. The temperature of the BSC was recorded overnight to establish the baseline temperature without work being performed and the instrument off (Supplementary Figure S3). Temperature data were downloaded off the datalogger and exported into Microsoft Excel and GraphPad Prism for analysis.

The CytExpert analyzer software v2.4 (Beckman Coulter, Brea, CA) assesses temperature of the lasers in real time. During six experiments, the temperature of the UV laser was recorded directly from the instrument software immediately prior to QC bead acquisition. The UV laser temperature was recorded with the BSC off and on at 0-, 1-, 2-, and 4-h time intervals in degrees Celsius (°C).

### System Stability

System stability was first measured using a combination of the Daily QC beads and the IR QC beads to monitor multi-laser signal drift and signal integrity. Fluorescence output was measured by median fluorescence intensity (MFI), a metric used to observe sample fluorescence signal over time. The robust coefficient of variance (rCV) was utilized to monitor the precision of the instrument’s fluorescence detectors. Robust CV is a measurement of variation in the detected fluorescence units and resolution between the bright, dim, and background electronic noise signal. The rCV was utilized to eliminate the statistical impact of any outliers. The standard rCV failure cutoff in the CytExpert software is ± 5% variation. To measure signal changes and instability over the course of the experiment, 10,000 single bead events were collected with BSC off and BSC on at 0-, 1-, 2-, and 4-h time intervals with three replicates for each time point. To extract the MFI and rCV values, the QC and IR bead populations were gated as shown in Supplementary Figure S4.

Next, we assessed instrument’s ability to continuously acquire reliable and stable data over the course of five minutes, which reflects both fluidic and optical stability. Fluctuations were indicated by fluorescent signal changes over acquisition time. The instrument stability was measured by collecting multi-size and multi-intensity CS&T QC bead samples for five-minute intervals at a rate of 10 μL/minute. Each measurement was reproduced in triplicate. To capture the instrument stability over the course of the experiment, time course data points were collected with BSC off and BSC on at 0-, 1-, 2-, and 4-h time intervals. To extract the MFI and rCV values, the CS&T QC bead populations were gated as shown in Supplementary Figure S4.

### Aerosol Generation and Sampling

Aerosol production was evaluated based on the previously described Cyclex-D (Environmental Monitoring Systems, Charleston, SC) DB assay, except sampling sites were modified to target potential vulnerable aerosolization points.^9,10,17^ DB aerosols were sampled at four locations: tube loader, plate loader, waste container, and the sash directly outside the BSC. All locations were sampled in normal and failure simulation modes. “Normal” was defined as the analyzer working according to manufacturer’s recommendation with the waste container closed. “Failure” was defined as the analyzer working according to the manufacturer’s recommendation, but the waste container fitting loosened. Each testing site was sampled in triplicate and with the BSC off. In addition to the BSC off, the waste container and the sash were sampled with the BSC on. Two negative control sites, one on the lab bench outside the BSC and the other within the BSC approximately 19 inches from the sash and 4 inches above the instrument, were sampled to measure background. The negative controls were taken before the introduction of DB to the system. Positive control was generated from a BD FACSAriaIII cell sorter in failure mode as described.^10^ The cell sorter is in the same BSL-3 laboratory as the analyzer, so positive control collections were taken after sampling from the CytoFLEX LX was complete.

Cyclex-D cassettes connected by tubing to a vacuum pump (model 300700-A, Environmental Monitoring Systems, Charleston, SC) collecting at 20 L/min suction were used to capture aerosolized DB analyzed at 10,000–15,000 events/s for 10 min in tube mode. DB were analyzed in plate mode by adding 100–200 μL of DB to half of a 96-well polystyrene flat-bottom plate (Beckman Coulter catalog# 609844) and acquired after mixing each well for 1 s. The threshold was triggered off the B525 area channel. After acquisition, the vacuum pump was turned off, and the glass coverslips from the cassettes were transferred onto gridded glass microscope slides (Electron Microscopy Sciences, Hatfield, PA) and observed on a fluorescent microscope with camera and monitor (Leica Microsystems DMLB type 020–519.509, Wetzlar, Germany). Bead slides were read by two individuals for confirmation, and the mean bead count was calculated from those measurements.

### Data Analysis and Statistics

All FCS files (FCS 3.0) exported from the acquisition software were analyzed using FlowJo^™^ v10.7.2 and v10.8.1 Software (BD Life Sciences, Franklin Lakes, NJ). Data QC was performed using the FlowAI plugin version 2.3.1 (Becton Dickinson, Franklin Lakes, NJ), which utilizes R version R4.2.3 statistical programming software (R Core Team, Vienna, Austria).^21^ Statistics and figures were generated using GraphPad Prism software v9.1.1 (GraphPad Software LLC, San Diego, CA), Microsoft Excel 2008 (Microsoft, Redmond, WA), and BioRender (BioRender, Toronto, Ontario). Comparisons of quantitative variables among the different groups were performed using paired, two-tailed *t*-test or one-way Analysis of Variance (ANOVA) with a confidence level of 95% (α = 0.05; statistically significant *p*-value <0.05). Both multiple paired *t*-tests and paired *t*-tests were performed based on the data organization; the use of each test is indicated below.

## Results

### BSC Performance

Annual certification of every BSC is required within high-containment laboratories.^18^ The manufacturer of the BSC specifies the unit should meet the airflow requirements for certification as indicated in Supplementary Figure S2. The BSC was certified to these standards as shown in the certification table, with the exception that the exhaust flow required an alternate profile (Supplementary Figure S5). The tests in Supplementary Figure S5 highlight the certification measurements for the BSC airflow to maintain the manufacturer’s specified ranges and compliance with National Science Foundation/American National Standards Institute 49–2018.^18^ In addition, it is imperative that the BSC is certified with the instrument inside to demonstrate that the BSC performance is not compromised by instrument placement. Instrument placement within the BSC is detailed in Figure 1B. The certification results for this BSC-enclosed analyzer show the size of the BSC permits proper airflow around the instrument to allow laminar flow maintenance even though it permanently houses a cytometer.

### Both BSC and UV Laser Temperatures Remain Stable during Operation

**Figure 2.**
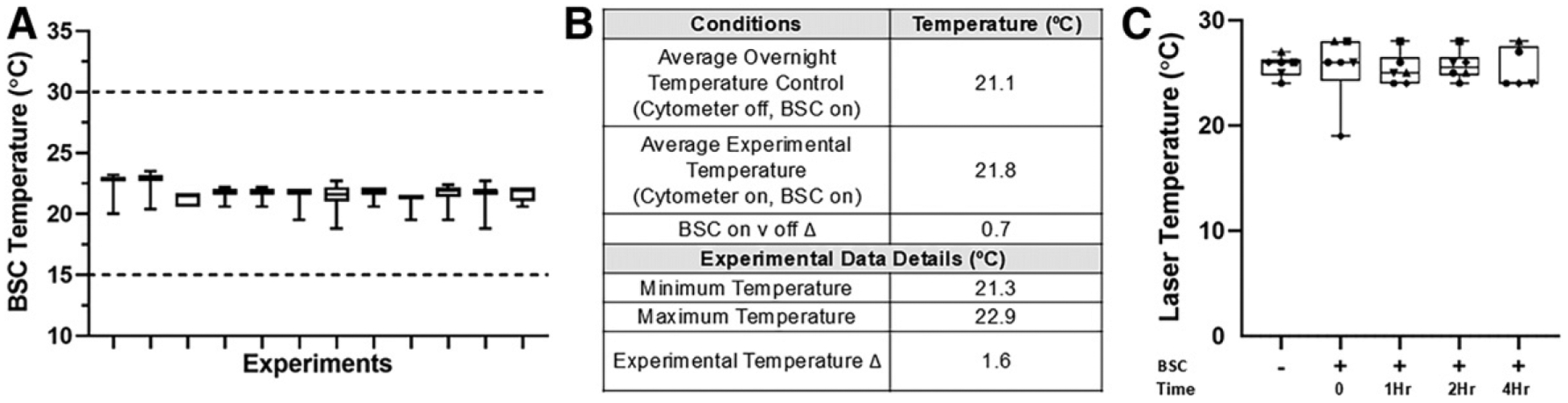
BSC and UV laser temperature stability. **(A)** Box and whisker plot showing the median temperature (°C) of the BSC during experimental days (*n* = 12). The BSC temperature is on the y-axis, and each experiment day is on the x-axis. The whiskers represent the min and max temperatures recorded and line within the box is the median temperature. Each box and whisker set represents one experiment with both the BSC off and on. The dotted lines at 15°C and 30°C represent the operating ambient temperature range according to the manufacturer. There is no statistical difference between the experimental days or the BSC off/on. This data excludes two days where the datalogger was recording overnight. This graph only shows the temperature logged during work in the BSL-3 and includes when the BSC was on and off. **(B)** Table depicting the average temperature of the BSC as measured by the datalogger to include the overnight logging when the instrument was off. For the “conditions” section, the temperature difference within the BSC between the instrument on and off is less than one degree. For the “experimental data” section, the minimum and maximum temperatures were recorded with the instrument and BSC on. All temperatures recorded fell well within the instrument’s ideal operational range. **(C)** The temperatures of the UV high photon energy laser was recorded over the span of several hours during six experiments (the 4-h point for five experiments). The X-axis shows the experimental time point and indicates if the BSC was on or off at the time point; (–) indicates the BSC was off, and (+) indicates the BSC was on. The Y-axis shows the UV laser temperature (°C). The upper and lower whiskers represent the minimum and maximum data point. If no upper/lower whisker is present, there are repeated minimum or maximum values. The center dividing line is the median of each time point. Each experiment is designated with a different symbol: circle is experiment 1, square is experiment 2, point up triangle is experiment 3, point down triangle is experiment 4, diamond is experiment 5, and hexagon is experiment 6. To test significance, an ordinary one-way ANOVA with Dunnett’s multiple comparison test (comparing each BSC on time point back with the BSC off) with an α = 0.05 was used. There was no statistical significance between any BSC on time point and the BSC off (all adjusted *p*-values greater than 0.05). UV, ultraviolet.

It is important to monitor the temperature of the analyzer’s environment, BSC heat contribution, and laser output to ensure it is operating within the manufacturer’s specified conditions. If the environmental temperature exceeds the recommended limit, multiple systems could be impacted, which would be reflected in the stability data. Figure 2A shows the variation in BSC temperature logged during 12 independent experiments.

Due to the enclosure of the analyzer within the BSC, we postulated that the BSC might contribute additional heat to the environment or not allow heat to dissipate. Baseline temperature measurements were taken inside the BSC with the BSC on and the instrument off over the course of two overnight monitoring periods. The average temperature inside the BSC with the instrument off was 21.1°C; the minimum temperature was 20.0°C and the maximum temperature was 22.7°C (data not shown). Figure 2B presents the experimental conditions tested and the average temperature for each condition. We compared the temperature of the BSC during the overnight logging when the instrument was off and during experiments when the instrument and BSC were on. The average temperature with the instrument off was 21.1°C, as previously observed, and the average temperature with the instrument on was 21.8°C, with a difference of 0.7°C. An average temperature change of less than 1°C is not enough to impact instrument stability and clearly shows the instrument is still operating within the optimal temperature range defined by the manufacturer.^22^ The average temperature logged with the instrument on during experimental periods was 21.8°C; the minimum temperature was 21.3°C and the maximum temperature was 22.9°C, with a delta (Δ) of 1.6°C (Figure 2B). While there were some temperature fluctuations, for these experiments the CytoFLEX LX was operated within the manufacturer’s specified ambient temperature range (15°C–30°C, and fluctuations < ±2°C/h) for the duration of all experiments.^22^ Overall, the temperature within the BSC remained stable over time during operations.

To assess laser performance, the temperature of the high-energy UV laser, which is the most sensitive laser in the system, was monitored throughout the experimental timeframe to look for temperature fluctuations. Laser temperature changes could be due to instrument heat output contained within the BSC or heat output from the BSC itself impacting the instrument. The temperature of the UV laser was recorded over the span of several hours (BSC off and BSC on at 0, 1, 2, and 4 h) during six experiments, except the 4-h time point was recorded for five experiments. The laser temperatures (°C) for each experiment and time point are outlined in Figure 2C. The temperature mean ± the standard deviation at each time point are as follows: BSC off 25.7 ± 1.0, BSC on at 0 h 25.5 ± 3.3, BSC on at 1 h 25.3 ± 1.5, BSC on at 2 h 25.7 ± 1.4, and BSC on at 4 h 25.4 ± 1.9 (data not shown). To test significance, an ordinary one-way ANOVA was performed. There was no statistical significance among means (*p* < 0.05). A Dunnett’s multiple comparisons test was performed to compare the mean of the BSC off time point (control) with the means of the BSC on time points. There was no statistical significance between any BSC on time point and the BSC off (α = 0.05 and all adjusted *p*-values greater than 0.05). Average temperature of the UV laser when the BSC was off and on found a less than 1°C difference. The average temperature of the UV laser when the BSC was off is 25.7°C and 25.5°C when the BSC was on, a difference of 0.2°C. We conclude the temperature of the UV laser is stable over time and the BSC is not impacting the laser temperature.

### Instrument Performance and Stability Are Preserved with BSC Enclosure under These Conditions

**Figure 3.**
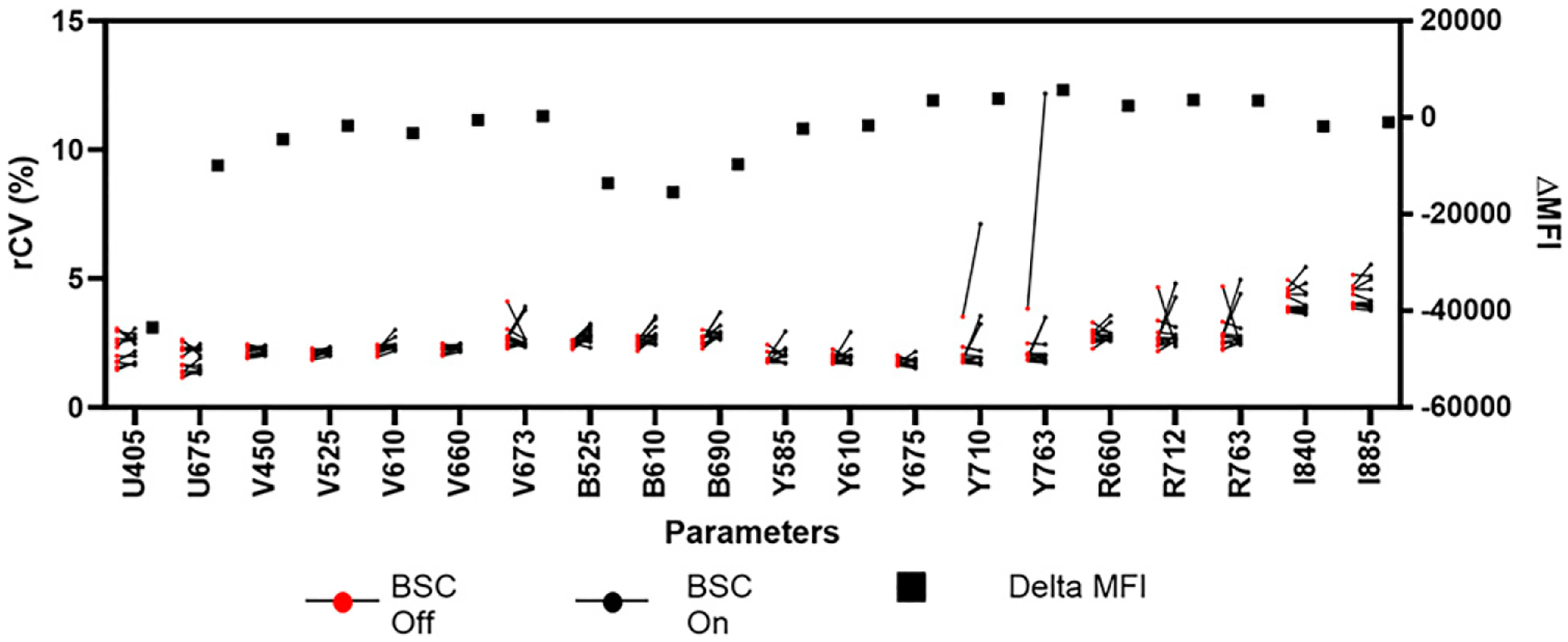
Combined graph showing the rCV on the left y-axis and represented by red (BSC off) or black (BSC on) dots. The solid lines between off and on dots connect the BSC status within each experiment day. The ΔMFI on the right y-axis is represented by black squares, by parameter. Daily QC and IR beads were used to generate this data. For the statistics, the rCV was averaged for each instrument parameter from 12 experiments. There was no statistical significance (adjusted *p*-values >0.05) of the rCV between BSC off and on. Multiple paired t-tests determined significance using the Holm-Sidak method with α = 0.05. ΔMFI of each parameter was calculated: average ΔMFI BSC on-average ΔMFI BSC off. There was no statistical significance (adjusted *p*-values >0.05) of the ΔMFI between BSC off and on. Multiple paired *t*-tests determined significance using the Holm-Sidak method with α = 0.05. IR, infrared; MFI, median fluorescence intensity; QC, quality control; rCV, robust coefficient of variance.

Measurements using the daily QC beads determined that the rCV remained consistent. Globally, there were no statistically significant changes across all the parameters (adjusted *p*-value >0.05) (Figure 3). There was some variation with the rCV values depending on the BSC status and the laser line. The average rCV values for the laser lines are blue 2.54% and 2.91%, red 2.81% and 2.95%, UV 2.06% and 2.09%, violet 2.31% and 2.38%, yellow 1.94% and 2.28%, and IR 4.21% and 4.22% for the BSC off and on, respectively (data not shown). Globally, the MFI of signal across all channels was very stable with both the BSC off and on using the daily QC beads. On average, the percent change of MFI with the BSC on was a 0.29% difference compared with the BSC off (data not shown). We did not observe a statistically significant change (adjusted *p*-value >0.05) in fluorescence with the BSC on versus BSC off (Figure 3).

**Figure 4.**
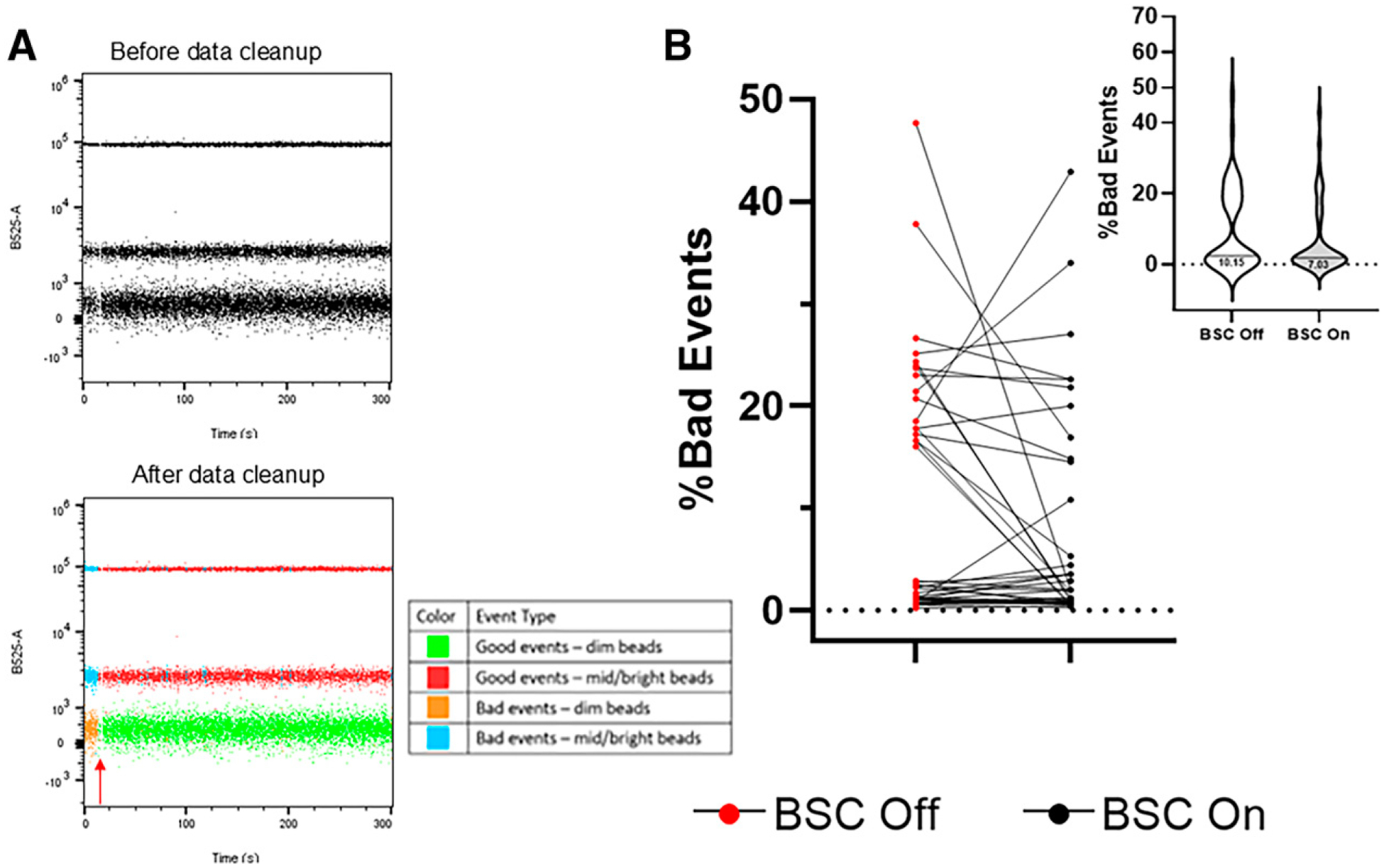
**(A)** Raw data prior and post FlowAI analysis showing an overlay of the bright, mid, and dim CS&T bead dot plots. The x-axis shows time (seconds), and the y-axis shows the signal intensity of a representative channel, in this case B525. Data from post FlowAI processing of the bright, mid, and dim bead populations; the plot shows the good and bad events overlayed. Blue and orange portions represent “bad events” and green and red portions represent “good events.” The red arrow is indicating a region of fluidic disruption and instability during the sample acquisition. **(B)** Scatter plot showing the % Bad Events comparing the BSC Off (red dots) with the BSC On (black dots). The solid lines between off and on dots connect the BSC status within each experiment day. Files were taken from 14 experiments. The frequency of bad events does not change in the presence or absence of the BSC, and there is no statistically significant difference between BSC Off and On (*p* = 0.075). This was calculated using a two-tailed paired t-test with an *n* = 42 for both BSC Off and BSC On (*n* = 42 total; 3 replicates from one time point in 14 experiments) with α = 0.05. The inset violin plot summarizes the %Bad Events distribution of the overall BSC Off and On data set. The solid black line and number represent the average %Bad Events for all BSC Off and On. CS&T, cytometer setup and tracking.

As another measurement of instrument stability, CS&T beads were collected at 5-min intervals and analyzed for fluidic disruptions using FlowAI (Figure 4A).^21^ The automatic computational tool analyzed each file and categorized each event as a “good event” or “bad event” according to the following factors: flow rate, signal acquisition, and dynamic range.^21^ The software then removed additional events based on abnormal signal acquisition and lower/upper limit of the instrument dynamic range.^21^ Based on the analysis of 42 BSC off and on files, on average 10.15% and 7.03% of events were classified as “bad” by the software for the BSC off and BSC on, respectively (Figure 4B). There is no statistical significance between the BSC off and BSC on “bad” files according to a two-tailed paired *t*-test (*p* > 0.05) (Figure 4B). Altogether, these data indicate the BSC did not significantly alter the stability of data collected over time.

### Aerosols Are Detected from the System

**Figure 5.**
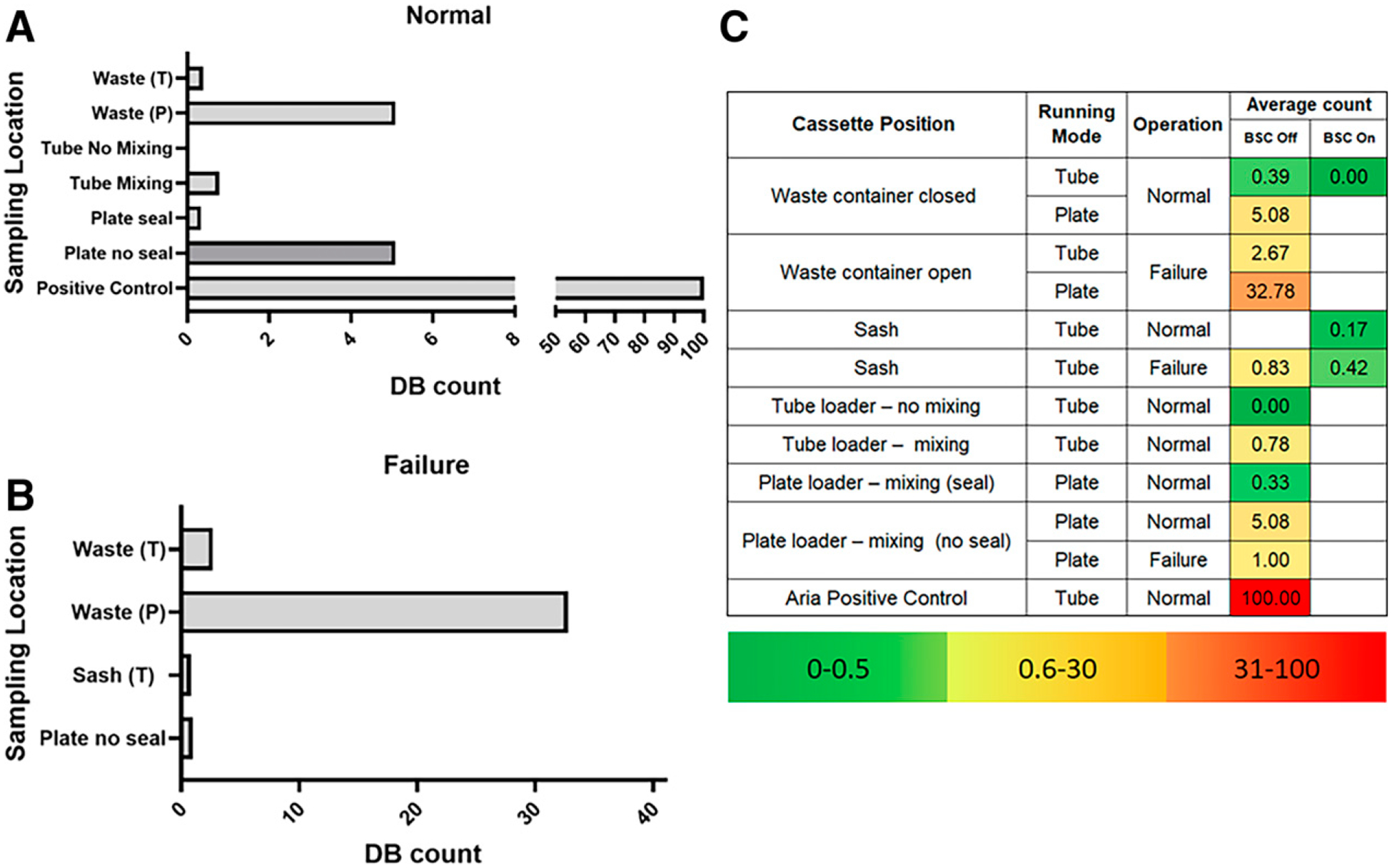
Aerosol results. (P) represents the plate acquisition, and (T) represents tube acquisition. **(A)** Average dragon bead (DB) count per slide with the BSC off in normal operation, defined as the waste container closed, grouped by sampling location. The average counts are as follows: Waste container during tube acquisition (0.39), Waste container during plate acquisition (5.08), tube no mixing (0), tube with mixing (0.78), plate with seal (0.33), plate no seal (5.08), and positive control (greater than 100). Ambient air from the lab bench before experiments were recorded (data not shown). **(B)** Average DB count per slide with the BSC off in failure operation defined as the waste container loosened. The average counts are as follows: Waste container with tube acquisition (2.67), Waste container with plate acquisition (32.78), Sash with tube acquisition (0.83), Plate no seal (1.00). **(C)** Heatmap of aerosol average DB counts where green indicates none or few beads, yellow indicates low number of beads, orange indicates medium number of beads, and red indicates high number of beads counted. A scale is provided beneath the table. Data are from between two and five experiments, and each location was sampled in triplicate. The table shows all positions and conditions sampled for DB aerosols and the average DB count. Running mode is the acquisition location of the DB (either tube or plate acquisition). Normal operation is the waste container closed, and failure operation is the waste container cap loosened. Blank spaces indicate that condition was not tested.

Aerosol detection is crucial for identifying containment vulnerabilities within the system. Using our previously published assay, aerosol-generating areas were confirmed at known weak points of the instrument.^17^ The negative control of ambient room collected on the lab bench and ambient air in the BSC did not show any DB (data not shown). The remaining sampling locations generated DB by the analyzer, particularly from the loosened waste container during failure mode, during plate acquisition, and tube acquisition with mixing. Under normal operations, on average we detected 5.08 DB from the plate loader with mixing, 0.78 DB from the tube loader with mixing, and no DB from the tube loader no mixing. These results show the plate loader and tube acquisition with mixing generate some aerosols even under normal operations (Figure 5A). Turning the automatic mixing feature off reduced the number of aerosols detected. To reduce the number of aerosols during plate acquisition, we used perforated plate sealer (X-Pierce^™^, Perkin Elmer, Waltham, MA) to test containment. We detected 0.33 DB and 5.08 DB with and without the plate seal, respectively. This demonstrates that a plate sealer could effectively minimize aerosolization during plate acquisition. In failure mode, it is notable that on average DB are detected at the waste container in both tube and plate acquisition, with a 12-fold increase in plate mode (2.67 and 32.78, respectively) (Figure 5B). DB were detected at low levels other than at the waste tank when testing failure mode. Beads were collected at the sash level during tube acquisition with mixing (0.83) and in front of the plate loader (1.00). Two locations were sampled with the BSC on and compared with the BSC off to show the BSC is effectively containing aerosols. With the conditions of BSC off and on, the closed waste container (0.39 and 0.00, respectively) and the sash (0.83 and 0.42, respectively) were tested in failure mode. The reduction in DB indicates that the BSC is effective and strengthens the support for an analyzer enclosure within a BSC. The sorter positive control met acceptance criteria of greater than 100 DB on the slide. Average DB counts are presented in Figure 5C.Supplementary Data S1 contains comprehensive, individual DB data.

## Discussion

Biosafety precautions are necessary when utilizing aerosol-generating lab instrumentation, but aerosols with flow cytometry analyzers have not been characterized to the same extent as cell sorters.^23^ We sought to assess the performance and containment of a benchtop analyzer fully enclosed within a Class II BSC. When placing an analyzer in a BSC, the following instrumental impacts should be considered: temperature fluctuations, system stability, and aerosol management. Frequent temperature changes can impact sample integrity and laser stability. System stability within the instrument ensures accurate MFI and rCV reporting. Although BSCs reduce potential aerosol exposure to both the operator and environment, there is a possibility the BSC could negatively impact data output quality. Properly characterizing the BSC impact can maintain data quality while greatly improving biosafety.

Our detailed characterization of the impact of BSC enclosure on instrument performance indicated that despite some variation in BSC temperature, the BSC did not have an impact on the stability of the signal or the performance of the cytometer during analytical acquisition under the tested conditions. When the instrument is powered on, the temperature within the BSC increases by an average of 0.7°C (from 21.2°C to 21.8°C); the minimum and maximum temperatures are 18.8°C and 23.5°C, respectively, which is within range of the manufacture’s recommended operating temperature (15°C–27°C).^22^ This would indicate that most samples would be unaffected by the small amount of heat generated or contained by the BSC. While the temperature is stable, heat-sensitive sample analysis spanning greater than 4 h might need to be externally monitored. Sensitive samples with prolonged exposure to heat can cause DNA denaturing, cell death, and protein degradation, which would affect data quality.^24^

Similarly, laser temperature remained stable for the duration of the experiments. In general, minor laser temperature fluctuations can present initially during the instrument warm-up time. For our purposes, the system start-up program and QC were completed prior to recording the laser temperature. The start-up program and QC align with the manufacture’s recommendations and encompass the UV laser warm-up time.^22^ The consistent UV laser temperature measurements demonstrate that there is no internal instrument or overall heat accumulation within the BSC. As ambient temperature increases, wavelength, light intensity, and laser beam shape are impacted and may cause unknown signal fluctuations.^25^ Therefore, laser performance is highly susceptible to external temperature variations, which could compromise stable laser temperature and reliable wavelength output.^26^ If the ambient temperature exceeds the recommended operational range for the instrument, there can be some wavelength changes that may impact fluorochrome excitation and data accuracy. While the other laser temperatures were not measured, the stable UV laser output and laser temperature stability ensure the longevity, signal integrity, and reduced laser output noise of the laser over time.^27^

The signal output was stable throughout the experiment regardless of the BSC off or on. There were no statistically significant changes between the BSC off and on reflected in the MFI and rCV values. The rCV cutoff for QC failure is about 5%, depending on the system, so the 0.8% rCV increase we observed overall would not be detectable during typical cell analysis. While the rCV values are not large enough to trigger a QC failure, the rCV should be closely monitored while the BSC is on to ensure any changes are not impacting the cell signal of dim or rare populations. For our purposes, daily QC and IR beads were used to generate consistent, repetitious experiments, but we recognize that beads do not behave similarly to cells. It would be beneficial to perform additional signal output testing utilizing cells, which will determine if the rCV and MFI values remain consistent with these findings.

The fluidic instability was observed inconsistently and could be eliminated with the use of data cleaning tools, such as FlowAI, so as not to affect downstream analysis. Importantly, data collected with the BSC on did not have any greater incidence of instability compared with BSC off, which indicates the operation of the BSC did not contribute to system instability. Some fluidic instability occurred within the first 20 s of acquisition, but it might be an instrument-specific anomaly. This instability could be mitigated by acquisition of the sample for 30 s prior to recording data. With small particle analysis, a lag period between acquisition and recording for fluidics is strictly required to achieve signal stability and reliable data collection. Indeed, initial fluidic instability has been observed with viromtery of H5N1 virus on this platform outside of a BSC.^28^ This further indicates that fluidic disruptions can be observed outside of the BSC under normal working conditions.^28^ We recommended close monitoring of fluidic instability through the use of data cleaning tools during analysis. Besides FlowAI, there are additional automatic data cleaning tools available that can serve similar functions with different stringencies, such as FlowClean, FlowCut, PeacoQC, or other packages.^29^

The aerosol results confirm previous observations and indicate that aerosols are generated at several vulnerable points in the system.^17^ In normal operations, aerosols generated and captured at the closed waste container, albeit at very low levels in tube mode, solidify the need for analyzer BSC containment. An increased number of aerosols were generated and detected at the plate loader, compared with tube acquisition. We next asked if simply affixing a plate seal could help reduce aerosols at the plate loader. We observed a 15-fold reduction in aerosol generation with the introduction of a plate seal. Closed waste tank aerosols were detected at high levels with the plate loader. Further study is needed to ascertain the reason behind the increased aerosols at the closed waste container in plate mode. While the plate loader has higher aerosol potential, utilizing an automatic sampler would reduce manual tube handling within the BSC. The presence of aerosols during normal operations reiterates the need for a BSC in case of instrument or containment failure, such as improper waste closure or sample line clog.

In system failure, tube and plate modes generate more aerosol at the loosened waste container. Plate mode generates more aerosols compared with tube mode, as shown by a 12-fold increase detected. To ensure a well-contained and safe system, the highly vulnerable waste tank should be tightly secured, and waste levels closely monitored throughout acquisition. The waste tank of the CytoFLEX LX is not fitted with an air output line. However, if the desired analyzer waste tank is fitted with an air output line, it is crucial to attach a filter to capture aerosols that could escape. In addition, tests were performed with the BSC on when in system failure. When directly compared with results with the BSC off, the BSC is effective in reducing the number of DB detected. Sampling at the sash is the closest proximal point to inside the BSC. At the sash with the BSC on, aerosols were detected in very low levels, which could be due to the force of the vacuum pump disrupting the air curtain. Both the DB reduction with the BSC on and detection of DB at the sash further strengthen the argument for analyzer enclosure in a BSC. It is important to note that the DB assay is not intended to be used as a validation of the BSC, but rather a measure of aerosol detection. The BSC is validated via standard certification methods independent of DB counting.

Although the BSC did not impact instrument operational parameters, we confirmed through aerosol containment studies the value of placing analyzers within a BSC when studying infectious samples. Our experience with enclosing an analyzer in a BSC demonstrates that the BSC does not impact analyzer performance. For laboratories considering placing an analyzer in a BSC, first, it is important to characterize each analyzer and BSC as a collective system so data impacted by the BSC enclosure can be documented. Second, laboratories should monitor system stability of the analyzer-BSC unit longitudinally to ensure data quality over time. However, enclosure might not be a universal solution due to space limitations or prohibitive costs. The laboratory can use alternative solutions to mitigate infectious aerosol risk, such as fixing samples prior to analysis. Individual laboratories should work closely with respective institutional biosafety departments to perform their own independent risk assessment and determine if enclosure is appropriate.

In alignment with the BMBL requirement for annual BSC recertification in high-containment laboratories, it is necessary to prepare the instrument for decontamination and preventative maintenance.^18^ To prepare the BSC-enclosed analyzer for decontamination, the instrument fluidic paths are decontaminated by running an appropriate disinfectant for a set contact time, such as 10% bleach for 30 min, followed by deionized water. Each laboratory should optimize a fluidic decontamination protocol that is effective for the infectious agent(s) handled and meets instrument manufacturer’s recommendations. Our detailed instrument decontamination procedures are outlined in Supplementary Data S2. Both the instrument and BSC are subsequently decontaminated with vaporized hydrogen peroxide (VHP), performed by an externally contracted company. Our experience with cell sorters and analyzers has shown VHP does not impact instrument performance and is the preferred decontamination method for our laboratory.

A limitation in our experiments involving the aerosol containment testing is the lack of standardized assays for analyzers; we took the gold-standard assay for cell sorters and adapted it for our purpose. This test has relatively low sensitivity, so depending on the individual laboratory setup and instrument, additional aerosol test points may need to be included to accurately capture instrument-specific aerosol point-of-exit. It is also important to acknowledge that a negative test does not prove absence of aerosol release. To generate a more uniform analyzer aerosol detection protocol, additional analyzer and BSC models should be assessed to verify the proposed methodology listed in this article. Despite the limitations of these findings, we believe the data can be translated into a meaningful framework for high-containment laboratories interested in performing live cell analysis on infectious samples and placing an analyzer into a BSC.

## Conclusion

A novel, BSC-contained flow cytometry analyzer as described could be considered for any research or clinical laboratory performing cell analysis of infectious samples. The BSC has minimal contribution to heat accumulation in the system but no measurable impact on the stability of the analyzer, with a negligible impact on the data. With growing numbers of novel infectious agents and necessity for single-cell-based technologies, flow cytometry analyzer placement in a BSC should be encouraged, especially in high-containment settings. The added level of containment would expand research capabilities for public health preparedness and biodefense threats without compromising data quality. In conclusion, we hope our findings can propel novel innovative solutions for infectious cell analysis and contribute to and ignite further investigations and standardization efforts surrounding flow cytometry and biosafety in high containment.

## Supplementary Material

Supplemental File 6

Supplemental File 7

Supplemental File 1

Supplemental File 2

Supplemental File 3

Supplemental File 4

Supplemental File 5

Supplementary Data S1

Supplementary Data S2

Supplementary Figure S1

Supplementary Figure S2

Supplementary Figure S3

Supplementary Figure S4

Supplementary Figure S5
