# Supplementary material for: Assessment of Biocontainment Efficacy and Flow Cytometric Impact of a Novel Platform in High Containment Laboratories
Source: Appl Biosaf. Author manuscript; Available in PMC 2026 Apr 22. (PMC13099074; doi:10.1177/15356760251378149)
Supplement: Supplemental File 6 [file NIHMS2158370-supplement-Supplemental_File_6.docx]

| **Cassette Position** | **Running Mode** | **Operation** | **Experiment 1** | | | | | | **Experiment 2** | | | | | | **Experiment 3** | | | | | | **Experiment 4** | | | | | | **Experiment 5** | | | | | |  |
| --- | --- | --- | --- | --- | --- | --- | --- | --- | --- | --- | --- | --- | --- | --- | --- | --- | --- | --- | --- | --- | --- | --- | --- | --- | --- | --- | --- | --- | --- | --- | --- | --- | --- |
|  |  |  | BSC Off | | | BSC On | | | BSC Off | | | BSC On | | | BSC Off | | | BSC On | | | BSC Off | | | BSC On | | | BSC Off | | | BSC On | | |  |
|  |  |  |  |  |  |  |  |  |  |  |  |  |  |  |  |  |  |  |  |  |  |  |  |  |  |  |  |  |  |  |  |  |  |
| Waste container closed | Tube | Normal | 1 | 0 | 0 |  |  |  | 0 | 1 | 0 |  |  |  | 1 | 0 | 0 |  |  |  | 0 | 2 | 0 | 0 | 0 | 0 | 0 | 0 | 0 | 0 | 0 | 0 |  |
|  |  |  | 0 | 0 | 0 |  |  |  | 1 | 0 | 1 |  |  |  | 2 | 0 | 0 |  |  |  | 0 | 2 | 0 | 0 | 0 | 0 | 0 | 0 | 0 | 0 | 0 | 0 |  |
|  | Plate |  | 0 | 2 | 19 |  |  |  | 1 | 1 | 0 |  |  |  |  |  |  |  |  |  | 6 | 24 | 3 |  |  |  | 0 | 4 | 1 |  |  |  |  |
|  |  |  | 1 | 2 | 19 |  |  |  | 1 | 0 | 0 |  |  |  |  |  |  |  |  |  | 4 | 25 | 4 |  |  |  | 0 | 4 | 1 |  |  |  |  |
| Waste container open | Tube | Failure | 0 | 2 | 1 |  |  |  | 5 | 2 | 7 |  |  |  |  |  |  |  |  |  |  |  |  |  |  |  | 0 | 0 | 0 |  |  |  |  |
|  |  |  | 2 | 4 | 1 |  |  |  | 8 | 1 | 15 |  |  |  |  |  |  |  |  |  |  |  |  |  |  |  | 0 | 0 | 0 |  |  |  |  |
|  | Plate |  |  |  |  |  |  |  | 74 | 48 | 74 |  |  |  |  |  |  |  |  |  | 21 | 32 | 30 |  |  |  | 1 | 0 | 3 |  |  |  |  |
|  |  |  |  |  |  |  |  |  | 95 | 31 | 74 |  |  |  |  |  |  |  |  |  | 33 | 33 | 37 |  |  |  | 1 | 0 | 3 |  |  |  |  |
| Sash | Tube | Normal |  |  |  |  |  |  |  |  |  |  |  |  |  |  |  |  |  |  |  |  |  | 0 | 0 | 0 |  |  |  | 1 | 0 | 0 |  |
|  |  |  |  |  |  |  |  |  |  |  |  |  |  |  |  |  |  |  |  |  |  |  |  | 0 | 0 | 0 |  |  |  | 1 | 0 | 0 |  |
| Sash | Tube | Failure | 0 | 0 | 1 | 0 | 0 | 1 | 2 | 3 | 4 | 0 | 0 | 0 |  |  |  |  |  |  |  |  |  |  |  |  | 0 | 1 | 0 |  |  |  |  |
|  |  |  | 1 | 1 | 0 | 0 | 1 | 0 | 0 | 1 | 0 | 0 | 0 | 3 |  |  |  |  |  |  |  |  |  |  |  |  | 0 | 1 | 0 |  |  |  |  |
| Tube loader - no mixing | Tube | Normal |  |  |  |  |  |  |  |  |  |  |  |  |  |  |  |  |  |  | 0 | 0 | 0 |  |  |  | 0 | 0 | 0 |  |  |  |  |
|  |  |  |  |  |  |  |  |  |  |  |  |  |  |  |  |  |  |  |  |  | 0 | 0 | 0 |  |  |  | 0 | 0 | 0 |  |  |  |  |
| Tube loader - mixing | Tube | Normal | 0 | 0 | 4 |  |  |  | 0 | 2 | 0 |  |  |  | 0 | 0 | 1 |  |  |  |  |  |  |  |  |  |  |  |  |  |  |  |  |
|  |  |  | 0 | 0 | 4 |  |  |  | 0 | 0 | 0 |  |  |  | 1 | 1 | 1 |  |  |  |  |  |  |  |  |  |  |  |  |  |  |  |  |
| Plate loader - mixing (seal) | Plate | Normal |  |  |  |  |  |  |  |  |  |  |  |  |  |  |  |  |  |  | 0 | 0 | 1 |  |  |  | 1 | 0 | 0 |  |  |  |  |
|  |  |  |  |  |  |  |  |  |  |  |  |  |  |  |  |  |  |  |  |  | 0 | 0 | 1 |  |  |  | 1 | 0 | 0 |  |  |  |  |
| Plate loader - mixing (no seal) | Plate | Normal | 0 | 10 | 20 |  |  |  | 2 | 0 | 1 |  |  |  |  |  |  |  |  |  |  |  |  |  |  |  |  |  |  |  |  |  |  |
|  |  |  | 0 | 10 | 15 |  |  |  | 2 | 0 | 1 |  |  |  |  |  |  |  |  |  |  |  |  |  |  |  |  |  |  |  |  |  |  |
|  | Plate | Failure |  |  |  |  |  |  | 4 | 2 | 4 |  |  |  |  |  |  |  |  |  | 0 | 0 | 0 |  |  |  | 0 | 1 | 0 |  |  |  |  |
|  |  |  |  |  |  |  |  |  | 3 | 1 | 1 |  |  |  |  |  |  |  |  |  | 1 | 0 | 0 |  |  |  | 0 | 1 | 0 |  |  |  |  |

**Supplemental File 6.** Table of raw DB data from five experiments. This table shows the replicate data of triplicate sampling at each location as well as inter-operator variability in reading the slides. Cassette position, running mode, operation condition, and BSC on or off status are described for each data point. Greyed-out boxes represent conditions that were not tested. Each test was performed in triplicate measurements (represented by three points per condition tested). Two operators read each slide (represented by two numbers vertically for each reading).
