# Supplementary material for: Assessment of Biocontainment Efficacy and Flow Cytometric Impact of a Novel Platform in High Containment Laboratories
Source: Appl Biosaf. Author manuscript; Available in PMC 2026 Apr 22. (PMC13099074; doi:10.1177/15356760251378149)
Supplement: Supplemental File 7 [file NIHMS2158370-supplement-Supplemental_File_7.docx]

**Supplemental File 7: Instrument Decontamination Procedure. This document is our laboratory’s Standard Operating Procedure describing the daily decontamination of fluidic paths after running infectious samples, as well as the procedures we follow to prepare the instrument for annual lab shutdown.**

The following instrument decontamination procedures are approved for the infectious agents and work performed on this instrument. Other decontamination procedures may be appropriate for other systems based on risk assessment for specific pathogens and in consult with institutional biosafety personnel, institutional review board, and instrument manufacturer recommendations. Described below are daily decontaminations procedures for tube and plate modes as well as procedures for instrument and BSC Decontamination in Preparation for Annual Laboratory Shutdown.

1. **Daily Decontamination: Tube mode**

Perform this procedure after running infectious samples in tube mode.

1. After running samples, the instrument sample path will need to be disinfected.
   1. Acquire 3 milliliter (ml) of 50% bleach in a 12x75 millimeter (mm) tube with a custom sample flow rate of 240 microliter (μL)/minute.
   2. Acquire 3ml of 10% bleach in a 12x75mm tube with a custom sample flow rate of 240 μL/minute.
   3. Acquire 3ml of deionized (DI) water in a 12x75mm tube with a custom sample flow rate of 240 μL/minute.
2. Perform a ‘Deep Clean’ procedure to decontaminate the flow cell and internal fluidic paths.
   1. Place the instrument in standby mode. The deep cleaning bottle should be full of 10% bleach.
   2. Select ‘Deep Clean’ in the Cytometer menu. The software message will ask if you want to start a deep clean. Click yes.
   3. When the deep clean process is finished, wait 30 minutes so that the 10% bleach contacts the flow cell and internal tubing. Select ‘ok’ from the pop-up window in the software.
   4. Select ‘Prime’ in the cytometer menu. Click ‘yes’ when the dialog box appears.
   5. Remove the deep clean bottle containing 10% bleach and dispose of the bleach down the lab sink drain. Rinse bottle with DI water.
   6. Install a deep clean bottle containing DI water. Perform another deep clean procedure. Allow the water to remain in the flow cell for at least 5 minutes.
   7. Select ‘Prime’ in the cytometer menu. Click ‘yes’ when the dialog box appears.
   8. Remove the deep clean bottle containing DI water and dump the excess DI water down the lab drain. Install a deep clean bottle containing 50% Contrad.
   9. Select ‘Prime’ in the cytometer menu. Click ‘yes’ when the dialog box appears.
   10. Run the ‘Daily Clean’ for 5 minutes of both Coulter Clenz and DI water.
   11. Re-install the right-side cover.
3. Using a gauze pad soaked with 10% bleach, wipe the sample wash station and mixer where the top of sample tube could contact wash station when sample tube is loaded/unloaded.
4. If the instrument is not needed, switch off the CytoFLEX in the software.
5. Close the software and log off the computer.
6. **Daily Decontamination: Plate Loader**

Perform this procedure after running infectious samples using the Plate Loader.

1. After recording samples in Plate Loader mode, create a cleaning plate by selecting ‘add plate’ in the plate layout window. Correctly select the plate type from the drop-down menu and select the first sampling sequence that reads the plate from left to right.
2. Set up your cleaning plate as follows:
   1. Fill wells A1-A12 with 250 μL of 50% bleach.
   2. Fill wells B1-B12 with 250 μL of 10% bleach.
   3. Fills wells C1-C12 with 250 μL of DI water.
3. Acquire 200 μL of 50% bleach with a custom sample flow rate of 240 μL/minute for wells A1-A12.
4. Acquire 200 μL of 10% bleach with a custom sample flow rate of 240 μL/minute for wells B1-B12.
5. Acquire 200 μL of DI water with a custom sample flow rate of 240 μL/minute for wells C1-C12.

*IMPORTANT NOTE: in plate mode, bleach/water wells must be run as a sample. Designation as ‘cleaning agent well’ or deionized water well’, will not allow to control volume or speed of the acquisition.*

1. Perform a deep clean using bottles of 10% bleach and DI water as described above in section A2.
2. Using a gauze pad soaked with 10% bleach, wipe the plate holder, door to plate loader, and area under the plate loader. Also wipe the tube holder.
3. Go to Cytometer > Daily Clean.
4. Prepare a 96-well plate with 3 wells containing 250 μL 10% bleach and 3 wells containing 250 μL water.
5. Select the appropriate plate type.
6. Select the appropriate wells in the plate layout.
   1. Highlight the wells with the 10% bleach and select ‘set at cleaning agent well’.
   2. Highlight the wells with the DI water and select ‘set as deionized water well’
   3. If the wrong well is accidently selected, highlight the well(s) and select ‘set at empty well’ to clear them.
7. Load the plate and press Start.
8. After the cleaning procedure remove the plate and click ‘Close’.
9. Go to Cytometer > Daily Clean.
10. Prepare a 96-well plate with 3 wells containing 250 μL Coulter Clenz and 3 wells containing 250 μL water.
11. Select the appropriate plate type.
12. Select the appropriate wells in the plate layout.
    1. Highlight the wells with the Coulter Clenz and select ‘set at cleaning agent well’.
    2. Highlight the wells with the DI water and select ‘set as deionized water well’
    3. If the wrong well is accidently selected, highlight the well(s) and select ‘set at empty well’ to clear them.
13. Load the plate and press Start.
14. After the cleaning procedure remove the plate and press Close.
15. Go to Cytometer > Sample Injection Mode > Semi-automatic Sampler.
16. The restart warning prompt appears on screen. Click ‘OK’.
17. Turn off the CytoFLEX using the software.
18. Turn the manual switch nob at the tube loading station from position P (plate) to position T (tube), which is a 90 degree turn counterclockwise.
19. Turn on the CytoFLEX using the software.
20. Ensure the sampler status icon located in the bottom right corner changes to display “Semi-automatic Sampler.”
21. If the instrument is not needed, switch off the CytoFLEX in the software.
22. Close the software and log off the computer.
23. **Instrument and BSC Decontamination in Preparation for Annual Laboratory Shutdown**

Perform this procedure when preparing the instrument for annual laboratory shutdown.

1. Ensure there is enough bleach in the waste tank for a final concentration of 10% when full.
2. Turn on the instrument and perform a startup and initialize according to manufacturer instructions.
3. Perform a fluidic decontamination procedure in semi-automatic mode as outlined above.
   1. Acquire 3ml of 50% bleach in a 12x75mm tube with a custom sample flow rate of 240 μL/minute.
   2. Acquire 3ml of 10% bleach in a 12x75mm tube with a custom sample flow rate of 240 μL/minute.
   3. Acquire 3ml of DI water in a 12x75mm tube with a custom sample flow rate of 240 μL/minute.
4. Perform a deep clean decontamination procedure as outlined above.
   1. Place the instrument in standby mode.
   2. Remove the right-side cover.
   3. Remove the deep clean bottle containing Contrad and install a deep clean bottle containing 10% bleach.
   4. Select Deep Clean in the Cytometer menu. The software message will ask if you want to start a deep clean. Click ‘yes’.
   5. When the deep clean process is finished, wait 30 minutes so that the 10% bleach contacts the flow cell and internal tubing. Select ‘ok’ from the pop-up window in the software.
   6. After 30 minutes, remove the deep clean bottle containing 10% bleach and dispose of the bleach down the lab sink drain. Rinse bottle with DI water.
   7. Install a deep clean bottle containing DI water. Perform another deep clean procedure. Allow the water to remain in the flow cell for at least 5 minutes.
   8. Remove the deep clean bottle containing DI water and dump the excess DI water down the lab drain. Install a deep clean bottle containing 5% Contrad.
   9. Select ‘Prime’ in the cytometer menu. Click ‘yes’ when the dialog box appears.
   10. Run the Daily Clean for 5 minutes of both Coulter Clenz and DI water.
   11. Install the right-side cover.
5. Turn off the instrument.
6. Gently mix the contents of the waste tank. Let sit for at least 30 minutes.
7. Using a gauze pad soaked with 10% bleach (or HypeWipes), wipe sample tube holder and wash station/mixer.
8. Wipe the BSC bench with 10% bleach.
9. Open as many areas of the instrument as possible by removing the front and side panels. This will allow the decontaminate such as vaporized hydrogen peroxide (VHP) to contact all areas within the instrument.
10. Prepare the computer and accessories.
    1. Wipe the keyboard and mouse 3 times with 10% bleach.
    2. Wipe the keyboard and mouse with water and 70% Ethanol.
    3. Shut down the computer.
    4. Wipe the outside of the computer and monitor with a paper towel soaked in 70% Ethanol. Be careful not to get the ports wet.
11. After 30 minutes, turn on the lab sink and let water run for a few minutes.
12. Disconnect the waste line from the waste container. Cap the waste container, wipe the outside of the waste container with a paper towel soaked in 10% bleach and remove from the BSC. Carefully transport to the lab sink. Once at the lab sink, carefully uncap the waste container and slowly pour contents down the drain. Allow the water to run for several minutes to flush the sink and drain.
13. Return the empty waste container to the BSC. Wipe the inside of the waste cap, waste probe, and outside of waste line sensor connections with a paper towel soaked in 10% bleach. Wipe all quick disconnects with a paper towel soaked in 10% bleach. Leave the waste tank empty and open to allow the VHP to enter.
14. Disconnect the sheath line from the sheath container. Cap the sheath container, wipe the outside of the sheath container with a paper towel soaked in 10% bleach and remove from the BSC. Carefully transport to the lab sink. Once at the lab sink, carefully uncap the sheath container and slowly pour any remaining contents down the drain. Allow the water to run for several minutes to flush the sink and drain.
15. Return the empty sheath container to the BSC. Wipe the inside of the sheath cap, and outside of sheath line sensor connections with a paper towel soaked in 10% bleach. Leave the sheath tank open to allow the VHP to enter.
16. Open as many parts of the instrument as possible to allow the VHP to enter. For example, remove the side panels and open the top cover.
17. The laboratory room, BSC, and instrument will be decontaminated by an external contractor using vaporized hydrogen peroxide (VHP). Once the institute’s biosafety department specified amount of time has passed, and the biological indicators placed during the decontamination show no signs of growth, then the lab can be cleared for annual BSC certification and instrument preventative maintenance.
18. During the CytoFLEX LX preventative maintenance make sure all components of the preventative maintenance kit are replaced, per the manufacture’s recommendation.
