# Supplementary material for: Assessment of Biocontainment Efficacy and Flow Cytometric Impact of a Novel Platform in High Containment Laboratories
Source: Appl Biosaf. Author manuscript; Available in PMC 2026 Apr 22. (PMC13099074; doi:10.1177/15356760251378149)
Supplement: Supplemental File 1 [file NIHMS2158370-supplement-Supplemental_File_1.docx]

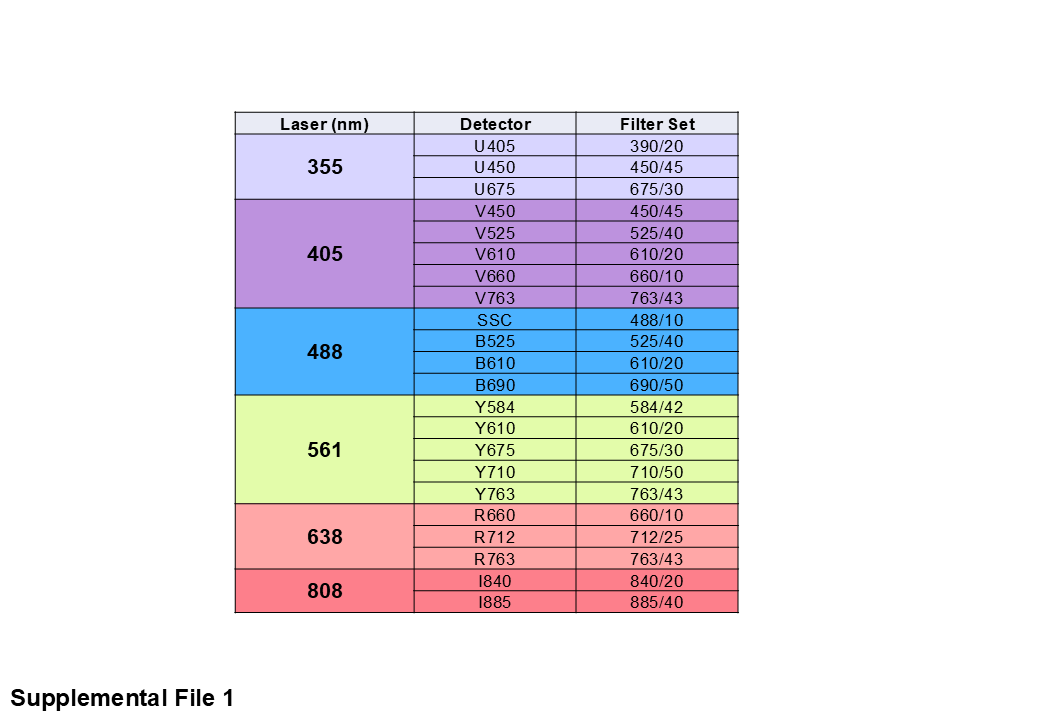


**Supplementary Figure S1**

**Supplemental File 1.** Instrument Configuration. The analyzer used in these experiments is a 6-laser, 22-channel CytoFLEX LX benchtop analytical flow cytometer. The instrument has the following lasers: 355 nanometer (nm) UV, 405nm Violet, Blue, 561nm Yellow-Green, 638nm Red, and 808nm Near-IR. The ‘detector’ column indicates the names of each detector within the instrument software. The ‘filter set’ column indicates the bandwidth range of each filter. For example, B525 filter optimally detects light at 525nm with ±20nm on each side of that wavelength so the total filter range for B525 is 505-545nm.
