# Supplementary material for: Assessment of Biocontainment Efficacy and Flow Cytometric Impact of a Novel Platform in High Containment Laboratories
Source: Appl Biosaf. Author manuscript; Available in PMC 2026 Apr 22. (PMC13099074; doi:10.1177/15356760251378149)
Supplement: Supplemental File 2 [file NIHMS2158370-supplement-Supplemental_File_2.docx]

.
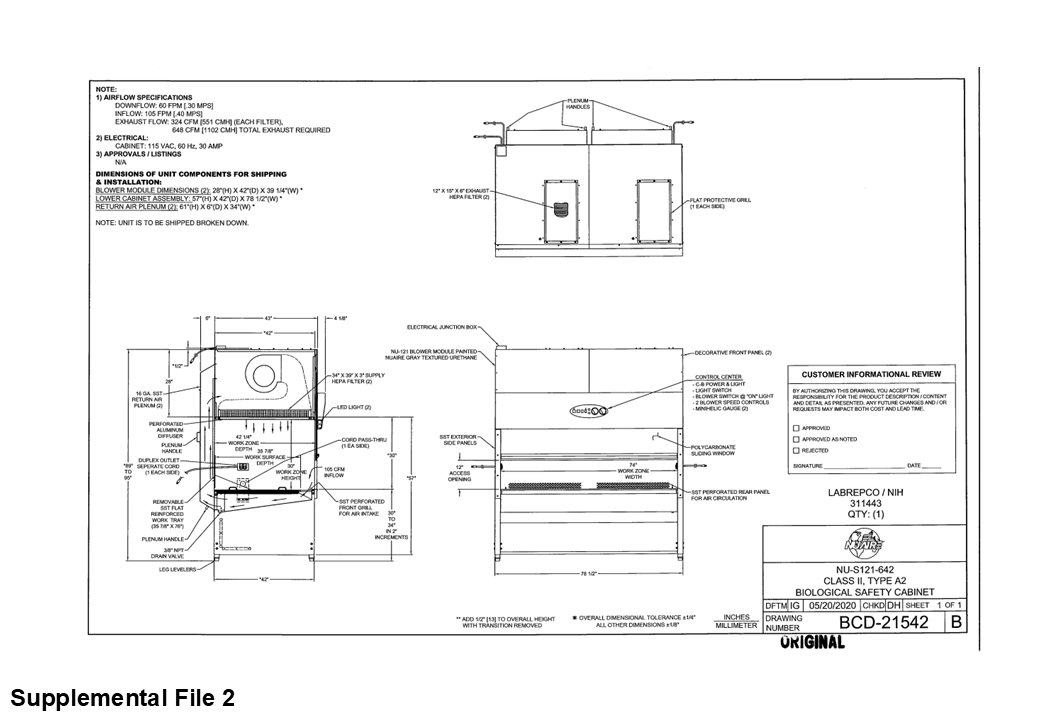


**Supplementary Figure S2**

**Supplemental File 2.** NuAire Biological Safety Cabinet Class II, Type A2 drawing from manufacturer. This schematic drawing shows the dimensions of the custom BSC workspace, and the dimensions of the overall unit. This drawing states the airflow and electrical specifications for the exact cabinet used for all experiments in the upper left portion of the drawing. Airflow specification abbreviations: FPM—feet per minute; MPS—meters per second; CFM—cubic feet per minute; CMH—cubic meters per hour.
