# Supplementary material for: Assessment of Biocontainment Efficacy and Flow Cytometric Impact of a Novel Platform in High Containment Laboratories
Source: Appl Biosaf. Author manuscript; Available in PMC 2026 Apr 22. (PMC13099074; doi:10.1177/15356760251378149)
Supplement: Supplemental File 3 [file NIHMS2158370-supplement-Supplemental_File_3.docx]

**
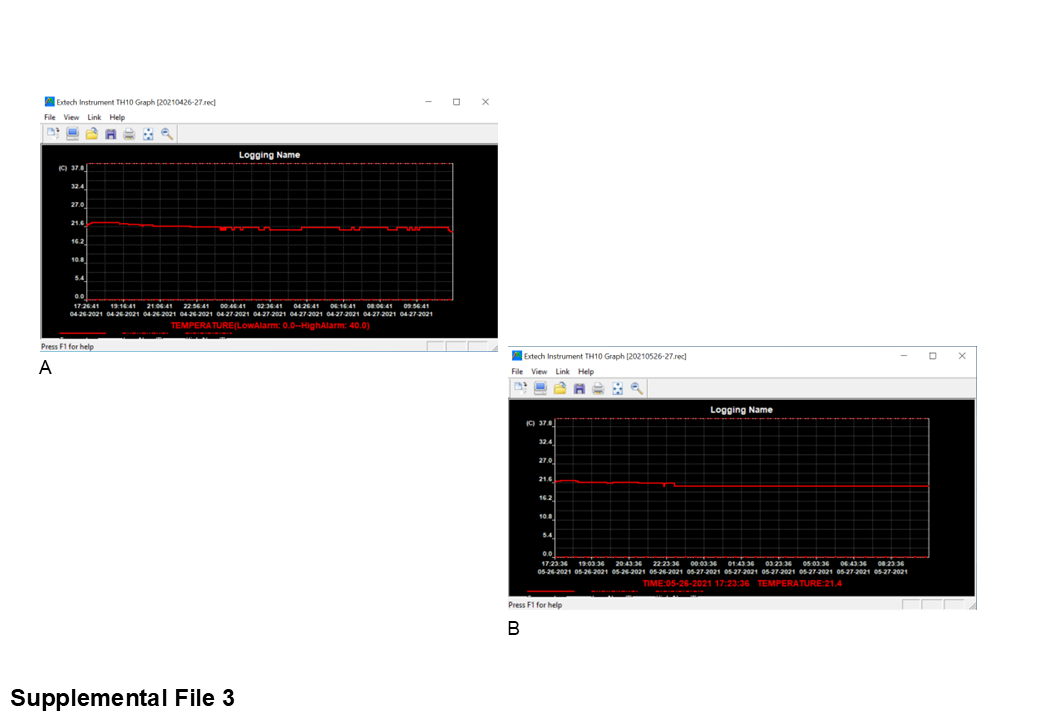
**

**Supplementary Figure S3**

**Supplemental File 3.** Extech datalogger logging output file from two overnight recordings where the instrument was off (A) and the BSC was on (B). The detection end of the datalogger was within the BSC. Temperature is on the y-axis and date and time is on the x-axis. The datalogger temperature range is 0°C to 40°C.
