# Supplementary material for: Assessment of Biocontainment Efficacy and Flow Cytometric Impact of a Novel Platform in High Containment Laboratories
Source: Appl Biosaf. Author manuscript; Available in PMC 2026 Apr 22. (PMC13099074; doi:10.1177/15356760251378149)
Supplement: Supplemental File 4 [file NIHMS2158370-supplement-Supplemental_File_4.docx]

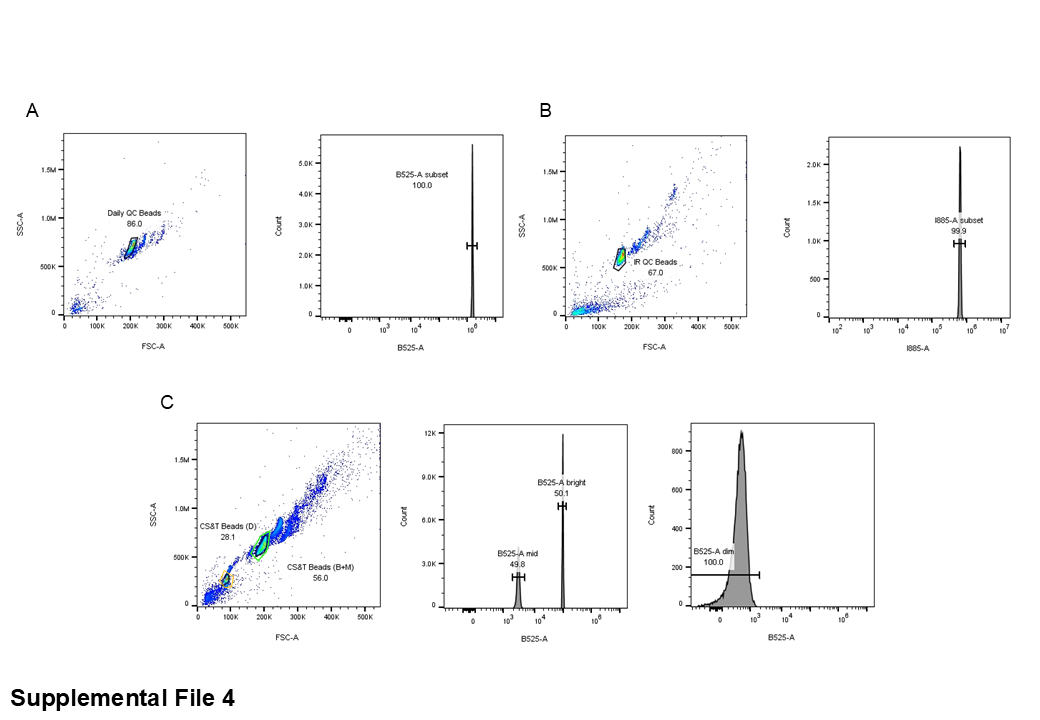


**Supplementary Figure S4**

**Supplemental File 4.** Representative dot plots and histograms showing the gating strategy used in data analysis. A) Daily QC Beads were gated on FSC-A vs SSC-A dot plot, then a histogram was generated for each channel and a histogram gate was set around the positive peak. B) Daily IR Beads were gated on FSC-A vs SSC-A dot plot, then a histogram was generated for each channel and a histogram gate was set around the positive peak. C) Multi-size CS&T beads were gated on a bright+mid (outlined in green) and dim (outlined in orange) populations based on FSC-A vs SSC-A, then a histogram was generated for each channel and gates set on the bright+mid and dim populations.
