# Supplementary material for: Assessment of Biocontainment Efficacy and Flow Cytometric Impact of a Novel Platform in High Containment Laboratories
Source: Appl Biosaf. Author manuscript; Available in PMC 2026 Apr 22. (PMC13099074; doi:10.1177/15356760251378149)
Supplement: Supplemental File 5 [file NIHMS2158370-supplement-Supplemental_File_5.docx]

**
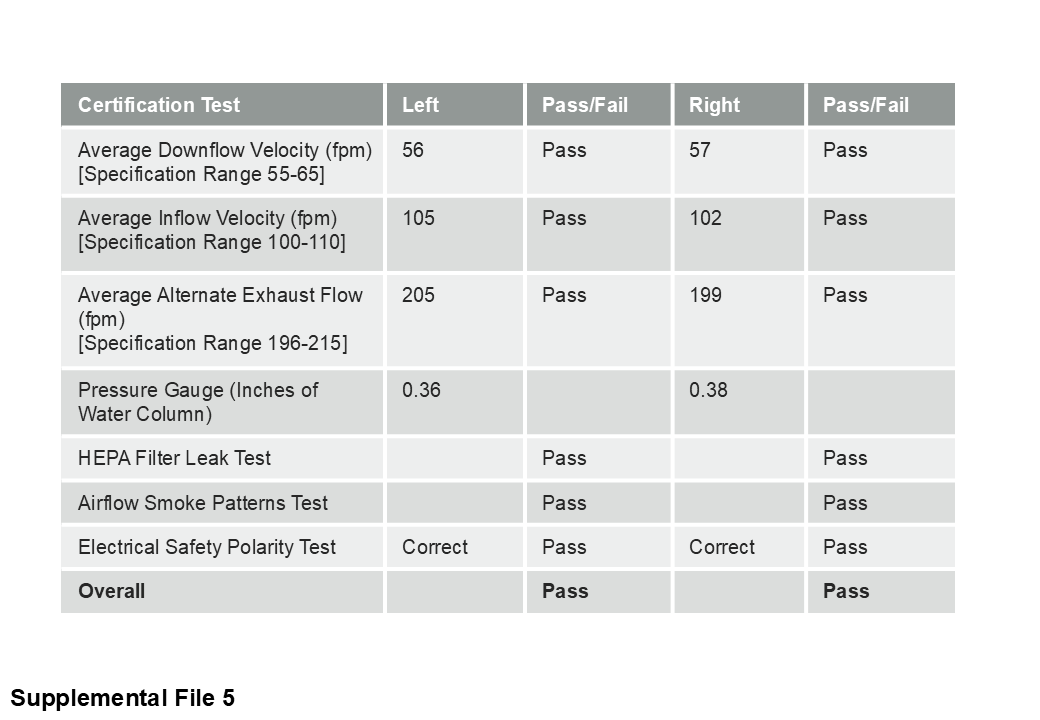
**

**Supplementary Figure S5**

**Supplemental File 5.** Summary of BSC certification results from initial BSC certification report. The BSC is a Class IIA cabinet and exhaust is vented to the room. Each side of the cabinet is certified independently as indicated by “left” and “right”. The Downflow Velocity Profile was uniform. The sash height was 12” for the Inflow Velocity Test. The certifier indicated the Exhaust Flow required an alternate exhaust profile. The average flow rates are presented along with indicating if the test passed or failed. The Exhaust Flow measurements were acquired in the unit feet per minute (fpm). The pressure gauge readings are also provided in inches of water column (“wc), as are additional tests performed during certification. Blank spaces indicate a measurement was not provided in the certification report and is a pass/fail result. Overall, each side of the BSC passed certification.

.
